# Locally Expansive Solutions for a Class of Iterative Equations

**DOI:** 10.1155/2013/372681

**Published:** 2013-11-05

**Authors:** Wei Song, Sheng Chen

**Affiliations:** Department of Mathematics, Harbin Institute of Technology, Harbin, Heilongjiang 150001, China

## Abstract

Iterative equations which can be expressed by the following form *f* ^*n*^(*x*) = *H(x*, *f(x)*, *f* ^2^(*x*),…, *f* ^*n*−1^(*x*)), where *n* ≥ 2, are investigated. Conditions for the existence of locally expansive *C* ^1^ solutions for such equations are given.

## 1. Introduction

Let *C*(*X*, *X*) be the set of all continuous self-mappings on a topological space *X*. For any *f* ∈ *C*(*X*, *X*), let *f* ^*m*^ denote the *m*th iterate of *f*; that is, *f* ^*m*^ = *f*∘*f* ^*m*−1^, *f* ^0^ = *id*,  *m* = 1,2,…. Equations having iteration as their main operation, that is, including iterates of the unknown mapping, are called iterative equations. It is one of the most interesting classes of functional equations [1–4], because it includes the problem of iterative roots [2, 5, 6], that is, finding some *f* ∈ *C*(*X*, *X*) such that *f* ^*n*^ is identical to a given *F* ∈ *C*(*X*, *X*). The well-known Feigenbaum equation *f*(*x*) = −(1/*λ*)*f*(*f*(*λx*)), arising in the discussion of period-doubling bifurcations [7, 8], is also an iterative equation.

As a natural generalization of the problem of iterative roots, iterative equations of the following form

(1) λ1f(x)+λ2f2(x)+⋯+λnfn(x)=F(x),                  x∈I=[a,b]

are known as polynomial-like iterative equations. Here, *n* ≥ 2 is an integer, *λ*_*i*_ ∈ ℝ  (*i* = 1,2,…, *n*), *F* : *I* → ℝ is a given mapping, and *f* : *I* → *I* is unknown. As mentioned in [9, 10], polynomial-like iterative equations are important not only in the study of functional equations but also in the study of dynamical systems. For instance, such equations are encountered in the discussion on transversal homoclinic intersection for diffeomorphisms [11], normal form of dynamical systems [12], and dynamics of a quadratic mapping [13]. Some problems of invariant curves for dynamical systems also lead to such iterative equations [14].

For the case that *F* is linear, where (1) can be written as

(2) $$\begin{array}{c} \lambda_{n}f^{n}\left(x\right)+\lambda_{n-1}f^{n-1}\left(x\right)+\cdots +\lambda_{1}f\left(x\right)+\lambda_{0}x=0, \end{array}$$

many results [15–17] have been given to present all of its continuous solutions. Conditions that ensure the uniqueness of such solutions are also given by [18, 19].

For the case that *F* is nonlinear, the basic problems such as existence, uniqueness, and stability cannot be solved easily. In 1986, Zhang [20], under the restriction that *λ*_1_ ≠ 0, constructed an interesting operator called “structural operator” for (1) and used the fixed point theory in Banach space to get the solutions of (1). Hence, he overcame the difficulties encountered by the formers. By means of this method, Zhang and Si made a series of works concerning these qualitative problems, such as [21–24]. After that, (1) and other type equations were discussed extensively by employing this idea (see [25–31] and references therein).

On the other hand, great efforts have been made to solve the “leading coefficient problem” which was raised by [32, 33] as an open problem. The essence of solving this problem is to abolish the technical restriction *λ*_1_ ≠ 0 and discuss (1) under the more natural assumption *λ*_*n*_ ≠ 0. As mentioned in [34, 35], a mapping *f* is said to be locally expansive (resp., locally contractive) at its fixed point *x*_0_, if |*f*′(*x*_0_)|>1 (resp., 0 < |*f*′(*x*_0_)|<1). In 2004, Zhang [35] gave positive answers to this problem in local *C* ^1^ solutions in some cases of coefficients, but this paper only discussed the locally expansive case and the nonhyperbolic case. In 2009, Chen and Zhang [34] gave positive answers to this problem with more combinations between locally expansive mappings and locally contractive ones and combinations between increasing mappings and decreasing ones. The main tools used in the two papers above are Schröder transformation and Schauder fixed point theorem. In 2012, J. M. Chen and L. Chen [36] consider the locally contractive *C* ^1^ solutions of the iterative equation *G*(*x*, *f*(*x*),…, *f* ^*n*^(*x*)) = *F*(*x*), and some results on locally contractive solutions of [34] were generalized. In 2007, Xu and Zhang [37] answered this problem by constructing *C* ^0^ solutions of (1). Their strategy is to construct the solutions piece by piece via a recursive formula obtained form (1). Following this idea, global increasing and decreasing solutions [38, 39] were also investigated.

Motivated by the above results, we will consider the existence of locally expansive *C* ^1^ solutions for the iterative equation of the following form:

(3) $$\begin{array}{c} f^{n}\left(x\right)=H\left(x,f\left(x\right),f^{2}\left(x\right),\ldots ,f^{n-1}\left(x\right)\right), \end{array}$$

where *n* ≥ 2. Some results on locally expansive solutions in [34] are generalized.

### 1.1. Basic Assumptions, Definitions, and Notations

Firstly, we state some assumptions on the known function *H* and the solution *f*. Let *I*, *J* be two intervals in **R**, *k* ∈ **Z**_+_,  *m*, *n* ∈ **N**, and let *C* ^*k*^(*I* ^*m*^, *J* ^*n*^) denote the set of all *C* ^*k*^ maps from *I* ^*m*^ to *J* ^*n*^. It is well known that, for a compact interval *I*, *C* ^0^(*I*, **R**) is a Banach space with the norm ||*h*|| = sup⁡_*t*∈*I*_ | *h*(*t*)|,  *h* ∈ *C* ^0^(*I*, **R**) and *C* ^1^(*I*, **R**) is also a Banach space with the norm ||*h*||_1_ = max⁡{||*h*||, ||*h*′||},  *h* ∈ *C* ^1^(*I*, **R**).

For convenience, let *X* denote (*x*_0_, *x*_1_,…, *x*_*n*−1_) ∈ **R** ^*n*^ and *O* (0,0,…, 0) ∈ **R** ^*n*^, where *n* = 2,3,…. Let *H*_*i*_′(*X*) denote (∂*H*/∂*x*_*i*_)(*X*), where *i* = 0,1,…, *n* − 1.

The assumption on *f* is

(f1) *f* ∈ *C* ^1^(*I*, *I*),  *f*(0) = 0, where *I* is an interval to be determined.

Assumptions on *H* are

(H1) *H* ∈ *C* ^1^(**R** ^*n*^, **R**),  *H*(*O*) = 0;
(H2) ∑_*i*=0_^*n*−1^|*H*_*i*_′(*O*)|>1;
(H3) |*H*_*i*_′(*X*) − *H*_*i*_′(*Y*)|≤∑_*j*=0_^*n*−1^ *M*_*ij*_ | *x*_*j*_ − *y*_*j*_| in a neighborhood *V* of *O* ∈ **R** ^*n*^, where *M*_*ij*_ are nonnegative constants, *i*, *j* = 0,1,…, *n* − 1.

Define a set

(4) $$\begin{array}{c} ℋ=\left\{H\;|\;H:R^{n}\to R\;\;\text{satisfies}\;\;\left(\text{H}1\right),\left(\text{H}2\right),\;\text{and}\;\;\left(\text{H}3\right)\right\}. \end{array}$$

Let *δ* > 0,  *τ* > 0, and  *M* > 0 be three constants, and define a set

(5) 𝒜(δ,τ,M)={ϕ∈C1([−δ,δ],R) ∣ ϕ(0)=0,|ϕ′(x)|   ≤ϕ′(0)=τ,|ϕ′(x)−ϕ′(y)| ≤M|x−y|,  ∀x,y∈[−δ,δ]}.

The set *𝒜*(*δ*, *τ*, *M*) is nonempty and is a convex compact subset of *C* ^1^([−*δ*, *δ*], **R**).

For *c* ∈ **R**,  |*c* | >1,  *ϕ* ∈ *𝒜*(*δ*, *τ*, *M*), and *H* ∈ *ℋ*, we define two functions as follows:

(6) $$\begin{array}{c} \lambda^{\phi }\left(s\right)=H\left(\phi \left(c^{-n}s\right),\phi \left(c^{-n+1}s\right),\ldots ,\phi \left(c^{-1}s\right)\right),\\ \lambda_{i}^{\phi }\left(s\right)=\frac{\partial H}{\partial x_{i}}\left(\phi \left(c^{-n}s\right),\phi \left(c^{-n+1}s\right),\ldots ,\phi \left(c^{-1}s\right)\right), \end{array}$$

where *i* = 0,1,…, *n* − 1,  *s* ∈ [−*δ*, *δ*]. By the choices of *H* and *ϕ*, we have *λ* ^*ϕ*^(0) = 0 and *λ*_*i*_^*ϕ*^(0) = *H*_*i*_′(*O*), where *i* = 0,1,…, *n* − 1.

If the solution *f* of (3) can be expressed as *f*(*x*) = *ϕ*(*c*(*ϕ* ^−1^(*x*))) by the Schröder transformation, where *c* is a constant to be determined, then (3) can be reduced to the following auxiliary equation:

(7) $$\begin{array}{c} \phi \left(c^{n}s\right)=H\left(\phi \left(s\right),\phi \left(cs\right),\ldots ,\phi \left(c^{n-1}s\right)\right). \end{array}$$

If function *f* is a solution of (3), then we can differentiate the equation. In fact, we can get that the derivative *f*′(0) is a zero of the following polynomial:

(8) $$\begin{array}{c} P\left(x\right)=x^{n}-H_{n-1}^{\prime }\left(O\right)x^{n-1}-\cdots -H_{1}^{\prime }\left(O\right)x-H_{0}^{\prime }\left(O\right). \end{array}$$

We refer to the polynomial (8) as the characteristic polynomial of (3).

Finally, we give a basic lemma.

**Lemma 1**

Let *D* ⊂ **R** ^*m*^ be a convex open set, and let *a* = (*a*_1_,…, *a*_*m*_) and *a* + *h* = (*a*_1_ + *h*_1_,…, *a*_*m*_ + *h*_*m*_) belong to $\bar{D}$. If $f:\bar{D}\to R$ is continuous on $\bar{D}$ and differentiable on *D*, then there exists a *θ* ∈ (0,1) such that

(9) $$\begin{array}{c} f\left(a+h\right)=f\left(a\right)+\overset{m}{\underset{i=1}{\sum }}\frac{\partial f}{\partial x_{i}}\left(a+\theta h\right)h_{i}. \end{array}$$

## 2. Main Results

 Let *S*(*n*) = {1,2,…, *n* − 1}.

**Theorem 2**

Suppose that *H* ∈ *ℋ*. Suppose that there is a neighborhood *U* of  *O* ∈ **R** ^*n*^ that satisfies

(*A*_1_^+^) for all *X* ∈ *U*, *H*_0_′(*O*) ≥ *H*_0_′(*X*) ≥ 0;
(*A*_2_^+^) for all *X* ∈ *U* and all *i* ∈ *S*(*n*), *H*_*i*_′(*O*) ≥ *H*_*i*_′(*X*) ≥ 0.

Then, (3) has a locally expansive increasing *C* ^1^ solution near 0.

**Theorem 3**

Suppose that *n* is odd and *H* ∈ *ℋ*. Suppose that there is a neighborhood  *U* of *O* ∈ **R** ^*n*^ that satisfies

(*A*_1_^−^) for all *X* ∈ *U*, *H*_0_′(*O*) ≤ *H*_0_′(*X*) ≤ 0;
(*A*_2_^±^) for all *X* ∈ *U*, *H*_*i*_′(*O*) ≥ *H*_0_′(*X*) ≥ 0 for all odd *i* ∈ *S*(*n*) and *H*_*i*_′(*O*) ≤ *H*_*i*_′(*X*) ≤ 0 for all even *i* ∈ *S*(*n*).

Then, (3) has a locally expansive decreasing *C* ^1^ solution near 0.

**Theorem 4**

Suppose that *n* is even and *H* ∈ *ℋ*. Suppose that there is a neighborhood  *U* of *O* ∈ **R** ^*n*^ that satisfies

(*A*_1_^+^) for all *X* ∈ *U*, *H*_0_′(*O*) ≥ *H*_0_′(*X*) ≥ 0;
(*A*_2_^∓^) for all *X* ∈ *U*, *H*_*i*_′(*O*) ≤ *H*_*i*_′(*X*) ≤ 0 for all odd *i* ∈ *S*(*n*) and *H*_*i*_′(*O*) ≥ *H*_*i*_′(*X*) ≥ 0 for all even *i* ∈ *S*(*n*).

Then, (3) has a locally expansive decreasing *C* ^1^ solution near 0.

## 3. Proof of the Main Results

**Lemma 5**

Under the conditions of Theorem 2 (Theorems 3 and 4, resp.), there is a constant *c* > 1 (resp., *c* < −1 in both cases) and *σ* > 0 such that for arbitrary given *τ* > 0, (7) has a  *C* ^1^ solution *ϕ* on [−*σ*, *σ*] with *ϕ*(0) = 0 and *ϕ*′(0) = *τ*.

**Proof**

If *c* is real and (7) has a local *C* ^1^ solution *ϕ* with *ϕ*(0) = 0 and *ϕ*′(0) ≠ 0, then by differentiating the equation, we can see that *c* is a root of characteristic polynomial (8).

If hypotheses of Theorem 2 hold, the hypothesis (H2) implies

(10) $$\begin{array}{c} P\left(1\right)=1-\overset{n-1}{\underset{i=0}{\sum }}H_{i}^{\prime }\left(O\right)=1-\overset{n-1}{\underset{i=0}{\sum }}\left|H_{i}^{\prime }\left(O\right)\right|<0. \end{array}$$

But *P*(*x*)→+*∞* when *x* → +*∞*, and this means that *P* has a root *c* > 1. In the case of Theorems 3 and 4, *P* has a root *c* < −1. Since for both of the cases *c* > 1 and *c* < −1, 0 < |*c* ^−*n*+*i*^ | <1,  *i* = 0,1,…, *n* − 1 and *c* is a zero of (8), we have

(11) |c2n|−∑i=0n−1|Hi′(O)||c2i| =|cn|(|cn|−∑i=0n−1|Hi′(O)||ci||c−n+i|) >|cn|−∑i=0n−1|Hi′(O)||ci|=0.

The above inequality holds because of the choice of the sign of *H*_*i*_′(*O*),  *i* = 0,1,…, *n* − 1. This also means that 1 − ∑_*i*=0_^*n*−1^|*H*_*i*_′(*O*)||*c* ^−2*n*+2*i*^| > 0. Now, we can choose a constant *σ*_1_ > 0 such that the following statements are true;

(*A*_1_^*σ*^) holds on [−*σ*_1_, *σ*_1_], where *σ* ∈ {+, −};
(*A*_2_^*δ*^) holds on [−*σ*_1_, *σ*_1_], where *δ* ∈ {+, ±, ∓};
(H3) holds on [−*σ*_1_, *σ*_1_]^*n*^.

For a given *τ* > 0, let

(12) $$\begin{array}{c} K_{2}=\frac{\tau^{2}\sum_{i,j=0}^{n-1}M_{ij}\left|c^{-2n+i+j}\right|}{1-\sum_{i=0}^{n-1}\left|H_{i}^{\prime }\left(O\right)\right|\left|c^{-2n+2i}\right|}. \end{array}$$

Furthermore, we can choose a 0 < *σ* < min⁡{*σ*_1_, *σ*_1_/*τ*} such that for, for all *ϕ* ∈ *𝒜*(*σ*, *τ*, *K*_2_), we have

(13) $$\begin{array}{c} \phi \left(\left[-\sigma ,\sigma \right]\right)\subset \left[-\sigma_{1},\sigma_{1}\right]. \end{array}$$

Define a mapping *𝒢* : *𝒜*(*σ*, *τ*, *K*_2_) → *C* ^1^([−*σ*, *σ*], **R**) as follows:

(14) 𝒢ϕ(s)=λϕ(s)=H(ϕ(c−ns),ϕ(c−n+1s),…,ϕ(c−1s)),                     s∈[−σ,σ].

In order to show that *𝒢* is a self-mapping on *𝒜*(*σ*, *τ*, *K*_2_), we calculate

(15) $$\begin{array}{c} \frac{d}{ds}𝒢\phi \left(s\right)=\overset{n-1}{\underset{i=0}{\sum }}c^{-n+i}\phi^{\prime }\left(c^{-n+i}s\right)\lambda_{i}^{\phi }\left(s\right). \end{array}$$

Obviously, *𝒢ϕ*(0) = 0. Since *c* ^*n*^ = ∑_*i*=0_^*n*−1^ *H*_*i*_′(*O*)*c* ^*i*^, we have

(16) dds𝒢ϕ(0)=∑i=0n−1c−n+iϕ′(0)λiϕ(0)=ϕ′(0)c−n∑i=0n−1Hi′(O)ci=τ.

Moreover, for all *s* ∈ [−*σ*, *σ*], by *A*_2_^*δ*^, *δ* ∈ {+, ±, ∓}, we have

(17) |dds𝒢ϕ(s)|=|∑i=0n−1c−n+iϕ′(c−n+is)λiϕ(s)|≤∑i=0n−1|c−n+i||ϕ′(c−n+is)||λiϕ(s)|≤|c−n|(∑i=0n−1|ci||Hi′(O)|)ϕ′(0)=τ.

By (H3) and the choice of *ϕ*, we can get that

(18) |dds𝒢ϕ(x)−dds𝒢ϕ(y)| =|∑i=0n−1c−n+iϕ′(c−n+ix)λiϕ(x)−∑i=0n−1c−n+iϕ′(c−n+iy)λiϕ(y)| ≤∑i=0n−1|c−n+i|{|ϕ′(c−n+ix)||λiϕ(x)−λiϕ(y)|          +|ϕ′(c−n+ix)−ϕ′(c−n+iy)||λiϕ(y)|} ≤∑i=0n−1|c−n+i|    ×{τ2∑j=0n−1Mij|c−n+j|+|c−n+i||Hi′(O)|K2}|x−y| =(τ2∑i,j=0n−1|c−2n+i+j|Mij   +∑i=0n−1|c−2n+2i||Hi′(O)|K2)|x−y|.

By the definition of *K*_2_, we get that

(19) $$\begin{array}{c} \left|\frac{d}{ds}𝒢\phi \left(x\right)-\frac{d}{ds}𝒢\phi \left(y\right)\right|\leq K_{2}\left|x-y\right|. \end{array}$$

Summing up the above discussion, we get that *𝒢*(*𝒜*(*σ*, *τ*, *K*_2_)) ⊂ *𝒜*(*σ*, *τ*, *K*_2_).

Now, we will prove that *𝒢* is continuous. Considering *ϕ*, *φ* ∈ *𝒜*(*σ*, *τ*, *K*_2_), by Lemma 1 and *A*_2_^*δ*^, *δ* ∈ {+, ±, ∓}, we have

(20) ||𝒢ϕ−𝒢φ|| =sup⁡s∈[−σ,σ]|λϕ(s)−λφ(s)| ≤sup⁡s∈[−σ,σ]∑i=0n−1|Hi′(O)||ϕ(c−n+is)−φ(c−n+is)| ≤(∑i=0n−1|Hi′(O)|)||ϕ−φ||.

Furthermore, by (H3), we have

(21) ||dds𝒢ϕ−dds𝒢φ|| =sup⁡s∈[−σ,σ]|∑i=0n−1c−n+iϕ′(c−n+is)λiϕ(s)       −∑i=0n−1c−n+iφ′(c−n+is)λiφ(s)| ≤sup⁡s∈[−σ,σ]∑i=0n−1|c−n+i||ϕ′(c−n+is)λiϕ(s)−φ′(c−n+is)λiφ(s)| ≤sup⁡s∈[−σ,σ]∑i=0n−1|c−n+i|      ×{|ϕ′(c−n+is)λiϕ(s)−ϕ′(c−n+is)λiφ(s)|        +|ϕ′(c−n+is)λiφ(s)−φ′(c−n+is)λiφ(s)|} =sup⁡s∈[−σ,σ]∑i=0n−1|c−n+i|      ×{|ϕ′(c−n+is)||λiϕ(s)−λiφ(s)|        +|ϕ′(c−n+is)−φ′(c−n+is)||λiφ(s)|} ≤∑i,j=0n−1τ|c−n+i|Mij||ϕ−φ||+∑i=0n−1|Hi′(O)|||ϕ′−φ′||.  

Finally, let

(22) $$\begin{array}{c} E=\max\left\{\overset{n-1}{\underset{i,j=0}{\sum }}\tau \left|c^{-n+i}\right|M_{ij},\overset{n-1}{\underset{i=0}{\sum }}\left|H_{i}^{\prime }\left(O\right)\right|\right\}, \end{array}$$

and we get that

(23) $$\begin{array}{c} {||𝒢\phi -𝒢\varphi ||}_{1}\leq E{||\phi -\varphi ||}_{1}. \end{array}$$

Now, the continuity of *𝒢* is evident. By Schauder's fixed point theorem, there exists a *ϕ* ∈ *𝒜*(*σ*, *τ*, *K*_2_) such that *𝒢ϕ* = *ϕ*. This means that (7) with the chosen *c* has a *C* ^1^ solution on [−*σ*, *σ*] with derivative *τ* at 0.

**Proof of Theorems 2–4**

Let *ϕ* be the solution of (7) obtained in Lemma 5. By the continuity of *ϕ*′, we are able to choose a neighborhood *J* ⊂ *ϕ*([−*σ*, *σ*]) of 0 such that *ϕ* ^−1^ exists and is also *C* ^1^ on *J*. Without any loss of generality, we can assume that *J* = *ϕ*([−*σ*, *σ*]). Hence, *ϕ* : [−*σ*, *σ*] → *J* is a homeomorphism. Moreover, we can choose a neighborhood *I* ⊂ *J* of 0 which is so small that *c* ^*i*^ *ϕ* ^−1^(*x*)∈[−*σ*, *σ*] for all *x* ∈ *I*,  *i* = 1,2,…, *n*. Let *f*(*x*) = *ϕ*(*cϕ* ^−1^(*x*)) for *x* ∈ *I*. Clearly *f* is also *C* ^1^ and invertible on *I*. Moreover, all iterates *f* ^*j*^,  *j* = 1,2,…, *n*, are well defined on *I*, and *f* ^*j*^(*x*) = *ϕ*(*c* ^*j*^ *ϕ* ^−1^(*x*)),  *x* ∈ *I*. Obviously, we have *f*(0) = 0, *f*′(0) = *c*, and *f* is locally expansive. Finally, for any *x* ∈ *I*, we have

(24) H(x,f(x),f2(x),…,fn−1(x)) =H(ϕ(ϕ−1(x)),ϕ(cϕ−1(x)),    ϕ(c2ϕ−1(x)),…,ϕ(cn−1ϕ−1(x))) =H(ϕ(c−n(cnϕ−1(x))),ϕ(c−n+1(cnϕ−1(x))),    ϕ(c−n+2(cnϕ−1(x))),…,ϕ(c−1(cnϕ−1(x)))) =𝒢ϕ(cnϕ−1(x))=ϕ(cnϕ−1(x))=fn(x).

Therefore, *f* is a locally expansive *C* ^1^ solution of (3).

## 4. Examples

**Example**

Consider the following equation:

(25) $$\begin{array}{c} f^{3}\left(x\right)=2\sin\left(x\right)+\sin\left(f^{2}\left(x\right)\right). \end{array}$$

Obviously, *H*(*x*_0_, *x*_1_, *x*_2_) = 2sin(*x*_0_) + sin(*x*_2_). It is easy to verify that *H* satisfy the assumptions of Theorem 2. This equation has at least one locally expansive increasing *C* ^1^ solution in a neighborhood of 0.

**Example 2**

Consider the following equation:

(26) $$\begin{array}{c} f^{3}\left(x\right)=-2\sin\left(x\right)-\sin\left(f^{2}\left(x\right)\right). \end{array}$$

Obviously, *H*(*x*_0_, *x*_1_, *x*_2_) = −2sin(*x*_0_) − sin(*x*_2_). It is easy to verify that *H* satisfy the assumptions of Theorem 3. This equation has at least one locally expansive decreasing *C* ^1^ solution in a neighborhood of 0.

**Example 3**

Consider the following equation:

(27) $$\begin{array}{c} f^{4}\left(x\right)=2\sin\left(x\right)+\sin\left(f^{2}\left(x\right)\right). \end{array}$$

Obviously, *H*(*x*_0_, *x*_1_, *x*_2_, *x*_3_) = 2sin(*x*_0_) + sin(*x*_2_). It is easy to verify that *H* satisfy the assumptions of Theorem 4. This equation has at least one locally expansive decreasing *C* ^1^ solution in a neighborhood of 0.
